# *PharmReaDy*: A Longitudinal Platform for Industry Career Preparation for Student Pharmacists in the U.S.

**DOI:** 10.3390/pharmacy14010037

**Published:** 2026-02-13

**Authors:** Ashim Malhotra

**Affiliations:** Department of Pharmaceutical and Biomedical Sciences, California Northstate University College of Pharmacy, 9700 W Taron Drive, Elk Grove, CA 95758, USA; ashim.malhotra@cnsu.edu; Tel.: +1-916-686-8885

**Keywords:** workforce readiness, provost systems, industry pharmacy, pharmacist professional identity

## Abstract

As pharmacy career pathways diversify, professional doctoral programs such as PharmD face increasing pressure to demonstrate measurable workforce readiness outcomes within accreditation-constrained curricula. This study describes and evaluates *PharmReaDy*, a longitudinal, theory-informed workforce readiness platform embedded within a U.S. PharmD program. Guided by Tinto’s student retention framework, the platform integrates curricular, co-curricular, and experiential elements, including an industry-focused elective course, national professional competitions, targeted skills workshops, micro-credentialing opportunities, and experiential placements. Outcomes were assessed using enrollment trends, aggregate course evaluation data, academic performance indicators, and downstream participation in industry-aligned opportunities. Enrollment in the elective increased from 8 to 20 to 30 students across three offerings. Mean course evaluation scores across seven learning domains remained consistently high, ranging from 3.7 to 3.9 on a 4-point scale, with no statistically significant differences between cohorts (Welch’s *t*-tests, adjusted *p* > 0.05) and small positive effect sizes observed over time (Hedges’ *g* ≈ 0.20–0.29). Students demonstrated strong academic performance and increased participation in industry-focused competitions, scholarships, and post-graduate fellowship pathways. Findings from *PharmReaDy* indicate that workforce readiness can be meaningfully operationalized as a structured educational function embedded within professional curricula rather than being exclusively deferred to post-graduate training.

## 1. Introduction

In the United States, pharmacists’ professional identity is fragmented due to their myriad roles and responsibilities in varied professional settings [1]. Pharmacists’ professional identity formation deficiencies reinforce impaired workforce readiness, due to a paucity of workforce development programs aimed at fostering practice readiness for all pharmacy graduates [2]. Several factors contribute to compounding the complexities of designing and implementing workforce readiness programs for pharmacy students. Paramount among these is the U.S. practice to develop a “generalist” pharmacist workforce [3,4]. Other factors include (1) the presence of technical and knowledge super-specialties in pharmacy workforce settings, (2) the lack of matching in-house teaching expertise, (3) packed PharmD curricula, and (4) the cognitive and practice dissonance between one entry-level Doctor of Pharmacy (PharmD) degree for preparing a practice-ready pharmacy workforce for disparate professional settings [5,6,7,8,9].

Pharmacists in the U.S. work in community, hospital, compounding, and specialty pharmacies, such as nuclear pharmacies, and in retail, hospitals, nursing homes, hospice care facilities, research and development, MTM, academia, the government, the armed forces, and the pharmaceutical industry. Each field has its own subspecialties. While the U.S. Bureau of Labor Statistics projected a 5% growth in overall pharmacist jobs in the United States between 2023 and 2033, it is estimated that 3–5% of licensed pharmacists work in the pharmaceutical and biotechnology industry sectors [10]. This accounts for approximately 10,131–16,885 of the 337,700 registered pharmacy workforce as of 2023, working in some capacity in the industry pharmacy sector. However, working in the pharmaceutical industry necessitates a foundational understanding of the principles, workflow, and professional duties of various pharmaceutical industry operations and functions, including drug discovery and development, sales, marketing, regulatory affairs, medical affairs, and others. Many U.S. PharmD programs do not currently prepare graduates for these specialty areas, necessitating an urgent clarion call for pharmacy education to include academic and professional identity-forming curriculum and co-curriculum geared towards pharmaceutical industry jobs [11,12].

To address this challenge, here we report the design, implementation, and assessment of a novel incremental, longitudinal, and integrated workforce readiness program called *PharmReaDy. PharmReaDy* was formed as a portmanteau of “PharmDs Ready for Pharma.” The main goal of the *PharmReaDy* program was to prepare PharmD students while in school with the behaviors, abilities, knowledge, and skillsets, in other words, the “professional identity” and knowledge base needed for working in the U.S. pharmaceutical industry following degree completion. Grounded in Tinto’s Theory of Student Retention [13], combined with elements of Spiro’s cognitive flexibility theory [14], here we report the outcomes of an interactive program of cohort-based learning to facilitate working readiness for the pharmaceutical industry upon graduation.

Delivered through four sequential phases, instead of a standalone course in industry pharmacy, our cost-effective, adaptable and transferable *PharmReaDy* platform offers a stepwise comprehensive workforce readiness program to deliver the following five curricular design objectives: (1) starting from “hands-on” experiential activities of the Industry Pharmacists Organization (IPhO), (2) leading to a foundational Industry Pharmacy elective course, (3) resulting in industry-sector related co-curriculum, and (4) culminating in “Pharmaceutical Marketing” micro-credentialing and (5) IPPE and APPE rotations in the pharmaceutical industry, followed by the continuum of national competitive postgraduate fellowships and pharmaceutical industry residencies.

Abbreviations used include Doctor of Pharmacy (PharmD), Industry Pharmacists Organization (IPhO), Medical Science Liaison (MSL), Accreditation Council for Pharmacy Education (ACPE), Introductory Pharmacy Practice Experiences (IPPE), Advanced Pharmacy Practice Experiences (APPE), and medication therapy management (MTM).

## 2. Methods

### 2.1. The Educational Setting

The California Northstate College of Pharmacy offers a 4-year Doctor of Pharmacy (PharmD) degree, which is fully accredited by the Accreditation Council for Pharmacy Education (ACPE) for eight years. The college is in the vicinity of the San Francisco Bay Area, with many “Big Pharma” companies and smaller start-ups offering plenty of opportunities for training and employment of pharmacy students and graduates.

### 2.2. Need Determination—Student Focus Group and Exit Surveys

In 2020, the college conducted a career needs assessment of incoming PharmD students about the type of pharmacy settings they wanted to work in following graduation. Combining the findings of this student focus group and data from the 2019 and 2020 AACP Graduate Exit Survey, we found a significant shift in student interest from employment in the community pharmacy sector (63% interest in 2019 to 54% in 2020) to working in the pharmaceutical industry sector (5% of respondents expressing interest in 2019 compared to 17% in 2020). Our combined data demonstrated a 32% gain in student-expressed interest for readiness for the pharmaceutical industry workforce in the matriculating class of 2020. Based on these data, the Assistant Dean of Program Development and Accreditation envisioned and proposed to the College Curriculum Committee a comprehensive pharmaceutical workforce readiness program that would be offered through experiences and courses in a sequence of increasing complexity in a cohort-based format to students progressing from the P1 year to the P4 year in the PharmD program.

### 2.3. Design of the Comprehensive “PharmReaDy” Program

Table 1 outlines the design components of the *PharmReaDy* program. Briefly, this cohort-based incremental longitudinal program required students matriculating in the PharmD program to express interest and enroll in the program in the first semester of the 4-year PharmD program. Students enrolled in *PharmReaDy* engaged in a sequence of industry pharmacy co-curricular activities through specialized student organizations focused on the pharmaceutical industry, a formal elective course in Industry Pharmacy, seminars, micro-credentialing through certificates for becoming Medical Sciences Liaisons (MSLs), a specialized Pharmaceutical Marketing Certificate program, and specialized 6- to 12-week-long internships in our three partnering pharmaceutical companies. It was our deliberate intention to offer this program for the holistic “learn by doing” framework that was the backbone of *PharmReaDy.*

### 2.4. Curriculum, Instructional Design, Educational Frameworks, and Pedagogy

*Grounding in Tinto’s Student Retention Framework.* We carefully grounded our overall approach in Tinto’s Theory of Student Retention [9]. Specifically, in his seminal work, Tinto posits that relationships, collaboration, reliance, and interdependencies of learners with their peers, faculty, and institution form essential components of an overall support and student retention system. Table 1 lists the combination of several peer and institutional relationship-building strategies we embedded in *PharmReaDy* to inculcate student engagement, investment, and leadership. We emphasized student peer group-based interactions, such as the IPhO; peer-based group projects, such as preparing for IPhO’s VIP, with course-based learning; and expert-facilitated experiential learning programs, such as the Pharmaceutical Marketing Certificate and the industry rotations during IPPEs and APPEs. Spiro’s cognitive flexibility concepts are used by using repeated, pluralistic representation of the industry pharmacy in non-continuous, non-linear, “ill-structured” and complex learning by exposure to industry curriculum and co-curriculum.

*The P1 PharmReaDy VIP Project.* P1 students who expressed interest in enrolling in *PharmReaDy* were required to volunteer and join the college’s local chapter of the national IPhO. This entire project was operationalized from 2020 to 2023, and each year, approximately 18–20 students signed up to participate in teams for the VIP competition. We designed a stepwise program to onboard students and to familiarize them with drug discovery and development, as required for the IPhO VIP competition. The college developed a self-directed learning course in Drug Discovery and Development, made available to all interested students on Canvas. This course included an introduction to drug discovery principles, pharmacophore analysis, drug screenings, the use of bioinformatics in drug discovery, FDA regulatory basics, the FDA drug approval process, the phases of clinical trials, the abbreviated drug approval process, and the writing of the VIP project as a term paper within this supporting course. Two faculty members from the Department of Pharmaceutical and Biomedical Sciences served as faculty advisors and volunteered their time (~10 contact hours each semester in the P1 year) to help edit and enhance the student team’s proposal for VIP.

*The P2 Industry Pharmacy Elective Course, ELC785.* In 2020, in response to the need for teaching industry pharmacy practice identified by incoming and graduating students, as detailed above, the Assistant Dean of Program Development and Accreditation recruited industry experts. They consulted published literature to create the proposal for the first pharmaceutical industry-based elective course. The 2-credit unit course was structured across ten 2-h-long lectures taught by industry experts recruited by the Assistant Dean. In the first step, we recruited a Director of Regulatory Affairs, a Director of Medical Affairs, and a Co-Director of Marketing and onboarded them as adjunct faculty with the college. These faculty members worked as subject matter experts and developed the detailed curriculum, lecture materials, assignments, and grading rubrics for the assignments. The Assistant Dean, who was a full-time faculty member, provided logistical support, developed the syllabus, worked with the curriculum committee to obtain approval, and helped manage the Canvas course page, along with the grading components of the course. ELC785 was placed in the spring semester of the P2 year and was open to all students interested in industry pharmacy careers, especially those who expressed interest in and registered for the *PharmReaDy* program in the previous P1 year. Table 2 provides the list of topics taught in ELC785. Due to the nature of the involved teaching with subject matter experts who were employed in pharmaceutical companies in the San Francisco Bay area, ELC785 was a distance education course offered online from 2020 to 2023.

It should be noted that the topics in regulatory affairs and pharmacovigilance were included in detail in the elective course with deliberate intentionality. This is because these topics have emerged as knowledge and skill gaps in the pharmaceutical industry sector, based on industry trends forecasting.

*The P3 “Industry Careers Co-curriculum”.* In March 2020, student evaluations from the ELC785 course indicated that students would benefit from a parallel co-curriculum where they could learn about industry careers, prepare their CVs and cover letters, and complete other requirements for future employment while in pharmacy school. Students in the 2020 ELC785 course identified the Medical Science Liaison (MSL) career pathway as one of maximum interest. Between 2020 and 2021, the Assistant Dean of Program Development recruited volunteer industry pharmacy experts working in Medical Affairs as MSLs to deliver MSL-related introductory and preparatory sessions. We designed a trifecta of sessions delivered as consecutive synchronous virtual seminars in three expert subject areas, detailed in Table 3.

*The P3 Pharmaceutical Marketing Week and Certificate Program.* An “Industry Pharmacy Week” was implemented from 3–6 April 2023. The Industry Pharmacy Week had a comprehensive suite of activities for all P1–P4 students to help prepare PharmD students as the new workforce, as shown in Table 4. These activities focused on pharmaceutical marketing foundations and career paths and included four components. The design of this week was grounded in design thinking, as students had to retrieve the knowledge gained throughout the program and submit various projects by the end of the week related to pharmaceutical marketing.

### 2.5. Assessment and Data Sources

Program outcomes were assessed using a mixed-methods, program-evaluation approach incorporating multiple complementary data sources. Quantitative measures included elective course enrollment trends, aggregate course evaluation ratings across predefined learning domains, and academic performance indicators. Descriptive participation metrics were also tracked for industry-aligned co-curricular activities, including professional organization membership, national competitions, scholarship participation, and fellowship-related outcomes. Qualitative inputs consisted of instructor observations and structured review of student engagement artifacts generated through course assignments and co-curricular activities. All analyses were conducted using de-identified, aggregate data collected as part of routine educational assessment and program improvement.

### 2.6. Statistical Analysis

ELC785 Industry Pharmacy course evaluation data from 2021 (*n* = 6) and 2022 (*n* = 10) were analyzed using a combination of descriptive and inferential statistics. Because the cohorts consisted of independent student groups, comparisons between years were conducted using Welch’s independent-samples *t*-tests, which do not assume equal variances and are appropriate for small and unequal sample sizes. For each of the seven Likert-type scale items (4-point scale), we calculated means, standard deviations, and 95% confidence intervals of the mean differences between years. To account for the multiple comparisons across the seven items, Holm–Bonferroni corrections were applied to the resulting *p*-values to control the familywise error rate.

Effect sizes were estimated using Cohen’s *d*, with Hedges’ *g* reported as a small-sample bias correction, providing a standardized measure of the magnitude of observed differences even in the absence of statistical significance. Confidence intervals for mean differences were also reported to aid interpretation of both the direction and precision of effects. In addition to item-level analyses, items were conceptually grouped into broader domains (Course Design, Engagement, and Learning Outcomes) to permit higher-level interpretation of results.

Descriptive statistics were further summarized as composite means across all items for each year.

All analyses were conducted with an alpha threshold of *p* < 0.05 for significance testing. Given the small sample sizes, effect sizes and confidence intervals were emphasized as more robust indicators of practical significance. Visualizations were created using radar and heatmap plots to illustrate differences in item means between years and to highlight stability or trends across the domains evaluated.

## 3. Results

### 3.1. IPhO and VIP Outcomes

The number of students volunteering to join the college-level chapter of IPhO increased significantly from 6 in 2020 to 18 in 2023 (*p* < 0.01), demonstrating a substantial positive impact on student interest, engagement, and participation in organizations such as IPhO. While there is a cost associated with IPhO membership, since IPhO provided resources about industry pharmacy opportunities, this interest correlated strongly with students’ self-directed preparation and readiness for careers in the industry. Importantly, based on the guidance from college faculty advisors appointed to the college’s chapter for IPhO, and using the materials from the drug discovery course, ELC785, students collaboratively developed a hypothetical drug discovery project, defended their project in the national VIP competition, and won IPhO awards in two consecutive years. Furthermore, as explained below, the Assistant Dean of Accreditation, who led the industry pharmacy *PharmReaDy* program, negotiated with a pharmaceutical marketing company and obtained $5000 in student scholarships, which could partially offset the cost of membership, in addition to being applied towards tuition. These results are significant as they not only demonstrate that our approach is engaging and confidence-building, in accordance with Tinto’s Theory of Student Retention, but also that it provides independent national benchmarking regarding the quality of the content taught to the students.

### 3.2. Increase in Enrollment in the Industry Pharmacy Elective Course (ELC785)

ELC785 expanded substantially across three consecutive offerings, increasing from 8 students in the first year to 20 in the second and 30 in the third, representing a 275% cumulative growth in participation. This pattern paralleled an increasing interest in the *PharmReaDy* specialization overall and reflected the perceived value of industry-focused learning opportunities within the Doctor of Pharmacy curriculum.

### 3.3. ELC785 Course Evaluations and Impact on Student Self-Perception of Learning

**Figure 1:**
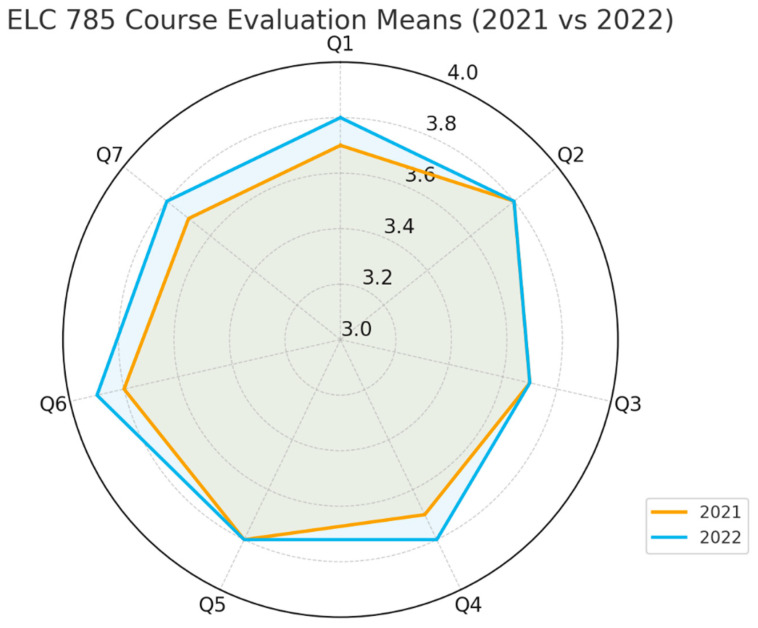
A radar plot showing the high and stable evaluation profile across all seven course evaluation items, with marginal outward extensions for the 2022 cohort in “Course Engagement,” “Level of Detail,” and “Advanced Understanding”. Q1–Q7 refer to the questions used in the student evaluation of the course.

Course evaluation data (Figure 1) were available for two academic years, 2021 (*n* = 6) and 2022 (*n* = 10). Mean ratings across all seven Likert-type scale items were consistently high in both years, ranging from 3.7 to 3.9 on a 4-point scale (equivalent to 92–98% agreement). Welch’s independent-samples *t*-tests revealed no statistically significant differences between the two cohorts after Holm–Bonferroni correction (all adjusted *p* > 0.05). Nonetheless, small positive mean shifts favored the 2022 cohort in items related to course engagement, level of detail, and advancement of understanding, with corresponding Hedges’ *g* effect sizes between 0.20 and 0.29, suggesting incremental improvement in perceived quality and content relevance.

**Figure 2:**
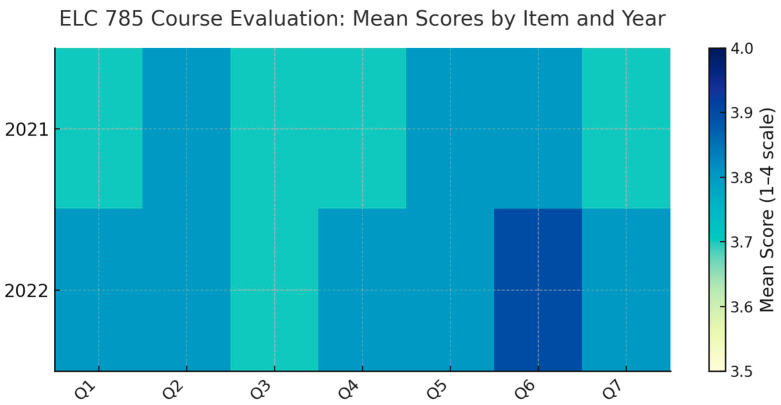
Student Course Evaluations for the Industry Pharmacy Elective course ELC 785 for 2021 (**top panel**) and 2022 (**bottom panel**). The student evaluation questionnaire solicits students’ evaluation regarding whether the course was engaging (Q1), contained an appropriate number of lectures (Q2), was well organized (Q3), had an appropriate level of detail (Q4), had enough opportunities to interact (Q5), and advanced student understanding of industry pharmacy (Q6) and whether online components such as expert guest lectures were comparable to the in-person lectures (Q7).

Complementing this, Figure 2 uses a heat map to illustrate the uniformity of mean responses across domains and highlights subtle year-to-year enhancements in key learning-outcome indicators. Together, these visualizations demonstrate that student perceptions of the elective remained exceptionally favorable and largely unchanged at near-ceiling levels, implying early stabilization of course quality and delivery despite an expanding enrollment base.

The composite means across all evaluation items increased modestly from 3.76 in 2021 to 3.80 in 2022, reinforcing the finding of consistent high satisfaction with the course structure, instructional design, and industry relevance. The absence of large statistical shifts was anticipated, given the small sample sizes and the already maximal scores achieved in the inaugural year. The aggregate data therefore suggest sustained excellence in teaching effectiveness and content design, with preliminary evidence of refinement and maturation of the elective as it evolved within the *PharmReaDy* framework.

## 4. Discussion

This discussion situates the *PharmReaDy* platform within the broader context of workforce readiness and professional identity formation in U.S. PharmD education. The sections that follow examine distinct but complementary implications related to longitudinal program design, alignment with industry career pathways, and the integration of curricular and co-curricular training.

### 4.1. PharmReaDy Addresses the Challenges of Designing and Assessing an Industry Pharmacy Professional Identity Workforce Readiness Program

Rather than functioning as a single curricular intervention, *PharmReaDy* operates as an integrated educational system spanning multiple years of professional training.

Professional identity is defined as a group’s self-identification and affiliation with the behaviors, aptitudes, and attitudes of a profession. Professional identity formation is a complex and multistage process that requires academic competence and continuous positive engagement with professional role models and career stalwarts [1]. Workforce readiness programs that simultaneously prepare pharmacy graduates for industry careers while helping sculpt an industry PharmD professional identity are challenging to design, implement, and assess. Jacob and Peasah note the paucity of pedagogical studies evaluating student pharmacists’ perceptions of the pharmaceutical industry during the first year of enrollment in the PharmD curriculum [15,16].

In accordance with the 2025 ACPE Standards [17], US PharmD programs prepare generalist pharmacists with a patient-centered focus. There are limited opportunities that prepare US pharmacy graduates for workforce readiness in the pharmaceutical industry, manufacturing, marketing, or compliance and quality control sectors [3,4,9]. Industry pharmacist professional identity-forming programs in PharmD programs remain rare. While industry pharmacy fellowships do exist, almost all exclusively occur following graduation, and have a competitive selection process resulting in a rate-limiting career gateway. This presents an urgent problem: industry career workforce readiness is compressed into postdoctoral residency or fellowship specializations with limited access instead of a comprehensive, longitudinal, and incremental development within the PharmD curriculum. Here, we present a cost-effective, adaptable, and theory-grounded solution to this pharmacy education problem. *PharmReaDy* shows the utility of a carefully designed and longitudinally integrated industry pharmacy curriculum and co-curriculum as a theory-grounded strategy for successfully augmenting acceptance into competitive industry fellowships following graduation.

### 4.2. PharmReaDy Fulfils an Academic, Curricular, and Co-Curricular Need for U.S. Industry-Pharmacy Trained PharmDs

Worldwide, many pharmacy programs include an industry pharmacy career focus; however, interestingly, in most Asian and African pharmacy schools, for example, if an industry focus is included, the curriculum and laboratory training typically emphasize formulation science, pharmaceutics, drug discovery, or engineering aspects [18]. This is markedly different from the typical entry point for US PharmD graduates, who would likely not be employed directly in formulation, a job typically reserved for research-oriented PhD careers. This creates an acute vacuum in the industry where a drug-expert PharmD-level workforce is needed to advise, consult, and operate non-manufacturing sectors.

*PharmReaDy* specifically addresses this critical gap by providing US PharmD programs with a tested and adaptable model. This manuscript details the step-by-step design, implementation, and assessment methodology and a platform for transferability to other US pharmacy schools. *PharmReaDy* includes flexible examples of assessment strategies that can help correct students’ learning trajectories by aligning them more specifically with industry needs. For example, *PharmReaDy’s* early inclusion of Medical Science Liaisons informed PharmD students about the significance of recognized MSL credentialing certificates and networking through LinkedIn, offering a unique advantage. Thus, *PharmReaDy* is a novel educational strategy applicable to most health profession education programs that lead to gainful vocational employment, targeting discipline-premised but non-research specialized positions in the pharmaceutical industry.

Evaluation of the limited education literature in industry pharmacy education shows that most papers report the scattered development of at most one industry pharmacy course as a specialized elective course of study, usually limited to an introductory course or rotation experience. This “one-off” inclusion of a technically complex and diverse pharmacy profession often does not engage professional PharmD students in immersive longitudinal exposure, which, according to Tinto, is an essential ingredient of a successful collegiate experience. As shown here, this curricular gap can be closed by adopting the *PharmReaDy* framework, which directly aligns pharmacy education with industry career readiness, reducing the time between graduation and industry entry.

### 4.3. PharmReaDy’s Theory-Informed Structure Keeps Learners Engaged

Specifically, the *PharmReaDy* framework offers a curricular bridge between undergraduate student experience and postgraduate readiness by functioning as a pre-fellowship readiness pipeline. The deliberate integration of a variety of co-curricular and curricular experiences, including national IPhO competitions, didactic courses, preparation of business plans, pharmaceutical marketing certificates, and industry pharmacy scholarships, is a seminal characteristic of Tinto’s student retention framework, allowing for the formation of long-lasting peer–peer, peer–faculty, and peer–industry mentor relationships. Incidentally, most students who elected to participate in PharmReaDy became members of IPhO in addition to joining other pharmacy professional organizations, such as APhA. Importantly, even if a US PharmD program faculty decided not to include an industry pharmacy focus for their school, *PharmReaDy’s* student engagement strategies offer important mechanisms to incentivize hands-on learning. This approach is further substantiated by Vardarajan et al. [19], who note that micro-credentialing, such as *PharmReaDy’s* pharmaceutical marketing certificate, is a “viable vehicle for the rapid upskilling of the workforce, offering potential pathways for gaining employment for some students.”

The conceptual design of *PharmReaDy* draws on prior theory-informed work addressing professional preparation in complex, ill-structured domains, including interprofessional education and high-stakes medicolegal practice under conditions of uncertainty [20,21]. Across these contexts, cognitive flexibility, role clarity, and professional identity formation emerge as central requirements for preparing trainees to function effectively within dynamic professional ecosystems. *PharmReaDy* applies these principles within industry pharmacy education by embedding longitudinal, multimodal experiences that support adaptive expertise and professional identity formation.

## 5. Limitations

While the *PharmReaDy* program offers a viable pathway for workforce readiness and industry pharmacist professional identity formation, our work is limited by the fact that it was implemented at a single institution and involved a relatively small number of self-selected PharmD students. These limitations are consistent with early-stage evaluations of educational innovations and underscore the need for future multi-institutional and longitudinal studies. Furthermore, even though our college is located in proximity to San Francisco, our strategic partnership with an East Coast pharmaceutical marketing company may be a viable strategy for pharmacy schools in areas of the U.S. that have fewer industry options.

## 6. Conclusions

*PharmReaDy* illustrates how an intentionally designed, longitudinal platform can embed workforce readiness and professional identity formation for PharmD industry careers within a professional PharmD curriculum without displacing core training requirements. By integrating curricular, co-curricular, and experiential elements within a coherent framework, the program translates early industry interest into measurable indicators of readiness and engagement. As pharmacy education continues to confront evolving workforce demands, such theory-informed and data-supported approaches offer a practical model for aligning professional education with emerging career pathways.

## Figures and Tables

**Table 1:** The Design Components of *PharmReaDy*.

Professional Year of Study (P1–P4 Year)	Name of the Learning Component	Type of Learning Activity	Faculty Facilitated (Y/N)	Number of Credit Units (c.u.) or Contact Hours (con. hr.)	Relationship Mechanism According to Tinto’s Theory
P1, fall and spring semesters	Required membership of the college chapter of the Industry Pharmacists Organization (IPhO)	Co-curriculum. Student cohort designs a proposal for the national U.S. IPhO student annual competition called the “Value of Industry Pharmacists” (VIP)	Y	27–30 con. hr.	Building peer support groups
P2, spring semester	Industry Pharmacy Elective Course, ELC785	Curriculum. A formal elective course introducing industry pharmacy operations with ten 2-h-long lectures and case-based presentations	Y Co-taught by 4 faculty, with 3 industry experts	2 c.u. (30 con. hr.)	Peer-collaboration, networking with industry professionals, commitment to career and graduation
P3, fall semester	Industry Careers Showcase Seminars—Pathway to MSL	*Co-curriculum.* A seminar series focused on industry career pathways and preparatory certifications for Medical Science Liaison careers	Y Co-led by industry experts recruited by the Asst Dean of Program Development	3–4 annual workshops (3–4 con. hr.)	Networking with industry MSLs, building institutional reputation
P3, spring semester	Pharmacy Marketing Certificate and the Pharmacy Marketing Week	Curriculum and Co-Curriculum. A specially designed collaboration between the college and a New Jersey-based Pharmaceutical Marketing Company, offering a 2-day comprehensive Pharmaceutical Marketing Certificate and a suite of seminars on pharmaceutical marketing, along with competitive student scholarships	Y Designed and hosted by the college’s Asst Dean of Program Development in partnership with the CEO of a pharmaceutical marketing company	16–18 con. hr.	Peer-collaboration, networking with industry experts, relationship building with the faculty, and the college.
P4, all semesters	APPE rotations. One or two consecutive specialty/elective APPE rotations	Experiential curriculum APPE rotations were developed in partnership with industry leaders in regulatory affairs, medical affairs, and marketing	Y	One 6-week-long residency-based APPE rotation or two consecutive residency-based APPE rotations (for 12 weeks)	Direct career mentoring, experiential learning, and institutional commitment

**Table 2:** Topics and Lectures in the Industry Pharmacy Elective Course, ELC785.

Week	Topic
1	Intro to Industry Pharmacy: Drug Development, Medical Affairs, and Commercial Aspects. Postgraduate opportunities in the pharmaceutical industry- Fellowships.
2	Overview of Pharmaceutical Industry and Clinical Development
3	Medical Affairs and the Scientific Platform
4	Medical Information and Medical Review
5	Introduction to Regulatory Affairs
6	Introduction to Pharmacovigilance
7	Clinical Operations: The role of Contract Research Organizations in the Pharma Industry
8	Pharmaceutical Sales
9	Business Development and the CNUCOP-EMBA perspective
10–13	Student Presentations
14	Final Exam

**Table 3:** The Industry Pharmacy Co-Curriculum—The MSL Career Pathway.

Session	Topic	Contact Hours	Assessment Method
1	Medical Science Liaison careers and industry fellowships	2	Student questionnaire
2	The MSL Society and the BCMAS and MSL-BC^®^ certification programs	2	
3	Developing and writing your CV, cover letter, and LinkedIn profiles to lan an MSL job	2	

**Table 4:** The Industry Pharmacy Week—Workforce Readiness Activities.

Session	Topic	Contact Hours	Assessment Method
1	Excellence in Industry Pharmacy Student Scholarships	2	Student questionnaire
2	The MSL Society and the BCMAS and MSL-BC^®^ certification programs	2	
3	Developing and writing your CV, cover letter, and LinkedIn profiles to lan an MSL job	2	
4	Using networking platforms to form connections for MSLs		

## Data Availability

The original contributions presented in this study are included in the article. Further inquiries can be directed to the author.
